# The Oncogenic Transcription Factor RUNX1/ETO Corrupts Cell Cycle Regulation to Drive Leukemic Transformation

**DOI:** 10.1016/j.ccell.2019.03.012

**Published:** 2019-04-15

**Authors:** Natalia Martinez-Soria, Lynsey McKenzie, Julia Draper, Anetta Ptasinska, Hasan Issa, Sandeep Potluri, Helen J. Blair, Anna Pickin, Asmida Isa, Paulynn Suyin Chin, Ricky Tirtakusuma, Daniel Coleman, Sirintra Nakjang, Salam Assi, Victoria Forster, Mojgan Reza, Ed Law, Philip Berry, Dorothee Mueller, Cameron Osborne, Alex Elder, Simon N. Bomken, Deepali Pal, James M. Allan, Gareth J. Veal, Peter N. Cockerill, Christian Wichmann, Josef Vormoor, Georges Lacaud, Constanze Bonifer, Olaf Heidenreich

## Main Text

(Cancer Cell *34*, 626–642.e1–e8; October 8, 2018)

During the preparation of the manuscript, we inadvertently left out Dr. Cameron Osborne, who was an important contributor to the chromatin capture experiments described in our article. His contribution is now included in the Author Contributions section, and his name has been added to the author list in the article online. The authors apologize for the omission.

